# Usefulness of using laser-induced photoacoustic measurement and 3.0 Tesla MRI to assess knee cartilage damage: a comparison study

**DOI:** 10.1186/s13075-015-0899-4

**Published:** 2015-12-30

**Authors:** Taku Ukai, Masato Sato, Miya Ishihara, Munetaka Yokoyama, Tomonori Takagaki, Genya Mitani, Yoshiki Tani, Tomohiro Yamashita, Yutaka Imai, Joji Mochida

**Affiliations:** Department of Orthopaedic Surgery, Surgical Science, Tokai University School of Medicine, Bohseidai, Isehara, Kanagawa 259-1193 Japan; Department of Medical Engineering, National Defence Medical Collage, 3-2 Namiki, Tokorozawa, Saitama 359-8513 Japan; Department of Radiology, Specialized Clinical Science, Tokai University School of Medicine, Bohseidai, Isehara, Kanagawa 259-1193 Japan

**Keywords:** Osteoarthritis, Diffusion tensor (DT) imaging, Laser-induced photoacoustic measurement (LIPA)

## Abstract

**Background:**

T2 mapping is an MRI method particularly reflective of the collagen arrangement in the cartilage, and diffusion tensor (DT) imaging captures the diffusion of water molecules. Laser-induced photoacoustic measurement (LIPA) makes it possible to assess not only the thickness of the cartilage layer but also its viscoelastic properties. By assessing cartilage damage assessment using LIPA and 3.0 Tesla MRI (T2 mapping and DT imaging), this study investigates the usefulness of the various methods.

**Methods:**

The International Cartilage Repair Society (ICRS) classification was used to classify 29 bone cartilage pieces excised during surgical procedures. At the same time, LIPA was performed at sites matching the area of cartilage damage. MRI was performed preoperatively to measure the T2 and the apparent diffusion coefficient. In addition, tissue sections for histological assessment using the Mankin score were prepared for each ICRS grade, and the results with the various methods were compared.

**Results:**

With DT imaging, significant differences were observed in all grades (*P* < 0.01). With T2 mapping, significant differences were observed in all grades except for grade 1 versus grade 2 (*P* < 0.01). With LIPA, significant differences were observed in ICRS grade 1 versus grade 3 (*P* < 0.05), grade 1 versus grade 4 (*P* < 0.01), grade 2 versus grade 4 (*P* < 0.01), and grade 3 versus grade 4 (*P* < 0.05). With the Mankin score, significant differences were observed in ICRS grade 1 versus grade 3 (*P* < 0.01), grade 1 versus grade 4 (*P* < 0.01), grade 2 versus grade 4 (*P* < 0.01), and grade 3 versus grade 4 (*P* < 0.01). Correlations were observed in all combinations of ICRS grade with DT imaging, T2 mapping, LIPA, and Mankin score. Correlations were observed between the degree of histological degeneration and DT imaging, T2 mapping, and ICRS grade, but LIPA had a weaker correlation than MRI.

**Conclusions:**

In the assessment of knee osteoarthritis, there are instances where it is difficult to assess the damaged cartilage site with MRI alone, and we believe that it is desirable to use a combination of LIPA and MRI.

## Background

The number of patients suffering from osteoarthritis (OA) of the knee has been estimated at 250 million people worldwide [1]. Although not life-threatening, OA of the knee reduces the activities of daily living and thus significantly reduces the patient’s quality of life. Osteophyte formation and joint space narrowing with X-rays are often used for diagnosing and assessing OA, respectively [2], but the essence of OA of the knee is a functional failure due to articular cartilage degeneration, and a lack of objective indices is due to the functioning of the articular cartilage itself. Moreover, because of the paucity of cellular components and the poor self-repair capacity of cartilage, it is important for OA to be both diagnosed and treated early. In recent years, cartilage has been assessed by magnetic resonance imaging (MRI), making it possible to visualize the soft tissue clearly for a multiplanar assessment. Standard MRI does not allow for assessment of early cartilage degeneration, but the latest MRI has provided a noninvasive means for biochemical assessment of the articular cartilage, including its glycosaminoglycans, collagen, and water content.

Of the forms of assessment by MRI, T2 mapping is regarded as enabling assessment of the water content and direction of collagen fibers in the cartilage, with its value reportedly being increased by damage to the cartilage matrix and especially by a decrease in collagen or an increase in water content [3]. Diffusion tensor (DT) imaging is a method where the phenomenon of water molecule diffusion is detected as a signal [4, 5], and it is already being used to assess spinal cord injuries [6] and cerebral infarction [7], but there are few reports on assessing cartilage damage [4, 5]. We have previously used DT imaging to assess cartilage damage that had been confirmed by arthroscopy and reported that it was possible to distinguish early cartilage damage up to advanced cartilage damage [8]. MRI is extremely useful in that cartilage damage can be assessed noninvasively, but it does not offer assessment of mechanical properties, such as the viscoelasticity properties, which represent a fundamental function of cartilage.

Irradiating the body with light or a laser produces, for example, an elevated temperature, fluorescence, or acoustic waves in association with scattering, reflection, and absorption [9, 10]. We have also focused on fluorescence and acoustic waves and reported that using a laser-induced photoacoustic diagnostic system (laser-induced photoacoustic measurement, LIPA) and time-resolved laser-induced fluorescence spectroscopy makes it possible to assess the thickness of the cartilage safely and to measure its viscoelasticity to assess its condition [11–23]. In this study, T2 mapping and DT imaging by 3.0 Tesla MRI were performed preoperatively to measure the T2 and the average diffusion coefficient (ADC). The International Cartilage Repair Society (ICRS) classification [24] was used to assess the extent of articular cartilage damage of bone cartilage pieces excised during total knee arthroplasty (TKA); at the same time, the relaxation time (τ) was measured using LIPA. Tissue sections of the bone cartilage pieces excised during surgery were prepared, and the usefulness of the various methods of examining cartilage damage was investigated by comparison with the results of Mankin’s histological scores [25] (Mankin score).

## Methods

### Patients

After approval from the research review committee of Tokai University School of Medicine, the present study was conducted with the written consent of two patients (one 74-year-old man and one 78-year-old woman (mean age 76 years)), each with one knee that had undergone TKA for OA of the knee at Tokai University Hospital between April and August of 2013.

### Surgical findings

The ICRS classification was used to classify 29 locations of the medial condyle of the femur, lateral condyle of the femur, posterior condyle of the femur, medial and lateral condyles of the tibia, and patellofemoral joint patella excised during surgery [24] (Table 1). LIPA was used to measure the τ for the cartilage lesions classified during surgery. For knee joint MRI, we performed T2 mapping and DT imaging on the day before surgery, and the T2 and ADC of the cartilage lesions confirmed during surgery were measured.

**Table 1:** International Cartilage Repair Society classification

Grade	Property
1	Superficial lesions, fissures and cracks, soft indentation
2	Defects that extend to less than 50 % in depth
3	Defects that extend to more than 50 % in depth
4	Complete loss of cartilage thickness, bone only

### MRI evaluation

The regions of interest (ROIs) were measured at all levels, from the cartilage surface to the deep zones, and the subchondral bone excluded carefully. Each ROI was measured within a range that measured 10 voxels high by 8 voxels wide [8, 26]. Three orthopedic surgeons and one radiologist measured the areas of cartilage damage on the MRI separately. To minimize disparities, the measurements were obtained three times, and the mean value was calculated.

### T2 mapping

T2 mapping was performed on an Achieva 3.0-T TX scanner (Philips Healthcare, Best, The Netherlands), with the patient’s knees positioned within a TX SENSE Knee eight-channel coil (Philips Healthcare). Imaging was conducted under the following conditions: sequence, multiecho turbo spin-echo; field of view (FOV), 120 × 120 mm; matrix, 211 × 320; repetition time (TR), 2510 ms; echo time (TE), 16, 32, 48, 64, 80, 96, and 112 ms; turbo factor, 7; slice thickness, 5 mm; gaps, 1 mm; number of excitations (NEX), 1; water-fat shift (WFS), 0.882 pixels/429.7 Hz; fat-suppression spectral presaturation with inversion recovery; and scan time, 8 min and 54 s [8].

### DT imaging

DT imaging was conducted under the following conditions: sequence, single-shot, spin-echo echo planar imaging (EPI); FOV, 150 × 150 mm; matrix, 144 × 144; TR, 2200 ms; TE, 68 ms; EPI factor, 73; number of slices, 13; slice thickness, 5 mm; gaps, 1 mm; NEX, 20; WFS, 28.628 pixels/15.2 Hz; fat-suppression spectral attenuated inversion recovery; MPG, 6; b-value, 600; half-scan factor, 0.678; and scan time, 10 min and 34 s [8].

### Data processing

From the DT imaging, the six components of the symmetric diffusion tensor were calculated [26]. For each voxel, the three eigenvalues (λ1, λ2, λ3) and their corresponding eigenvectors were calculated. The ADC was calculated from the eigenvalues as follows [27–29]:

$$ \mathrm{A}\mathrm{D}\mathrm{C}=\frac{1}{3}\left(\lambda 1+\lambda 2+\lambda 3\right). $$

### LIPA

Tissue viscoelasticity affects the propagation and attenuation of the stress waves induced by pulsed laser irradiation [11]. The relaxation time of the stress wave, calculated as the time in which the amplitude of the stress wave decreases by a factor of 1/e, gives the intrinsic relaxation parameter (h/G) of the tissue, where h is the viscosity and G is the elasticity. We have proposed a basic principle whereby the mechanical characteristics of the tissue can be measured using photoacoustic parameters. In this measurement technique, the relaxation time of the stress that acts on a linear viscoelastic object (consisting of a spring and a dashpot) is related to the viscoelastic parameters of the object and to the damping time of the stress waves generated by irradiation with a nanosecond pulse laser. Relaxation time is theoretically related to the viscoelastic ratio [9]. The relaxation time (τ) is calculated using the Levenberg–Marquardt algorithm, a nonlinear least-squares method, as follows. When the stress wave intensity is attenuated only by its reflection at the boundaries and its relaxation during its transmission through viscoelastic materials, then the time course of the stress wave intensity is expressed by the following equation [16]:

$$ {I}_{\delta }={I}_0\times R\times exp\left(-t\delta /\tau \right) $$

where *I*_0_ is the intensity of the stress wave at t = 0, R is the product of reflectivity (the product of the internal reflectivity at the interface at both ends of the sample), t_δ_ is the time after laser irradiation, and τ is the damping time of the stress wave that corresponds to the viscoelastic ratio.

### Histological assessment

For each form of cartilage damage, tissue sections were prepared from bone cartilage pieces excised during surgery. The sections were prepared by making cuts perpendicular to the tissue samples and fixed in 4 % paraformaldehyde for 1 week. After decalcification for 2 weeks using distilled water (pH 7.4) containing 10 % ethylenediaminetetraacetic acid, the tissue was embedded in paraffin wax and sectioned perpendicularly through the center of the cartilage damage. Each section was stained with Safranin O for glycosaminoglycans for histological evaluation [30]. For histological assessment, sections stained with Safranin O were assessed using the Mankin score [25] (Table 2). Three specialists of the Japanese Orthopaedic Association each separately performed the histological assessment. Each specimen was assessed, with 0 points as the lowest score and 14 points as the highest score.

**Table 2:** Mankin score for evaluation of articular cartilage degeneration

		Grade
I	Structure	
	a. Normal	0
	b. Surface irregularity	1
	c. Pannus and surface irregularity	2
	d. Clefts to transitional zone	3
	e. Clefts to radial zone	4
	f. Clefts to calcified zone	5
	g. Complete disorganization	6
II	Cells	
	a. Normal	0
	b. Diffuse hypercellularity	1
	c. Cloning	2
	d. Hypocellularity	3
III	Safranin–Orange staining	
	a. Normal	0
	b. Slight reduction	1
	c. Moderate reduction	2
	d. Severe reduction	3
	e. No dye note	4
IV	Tidemark integrity	
	a. Intact	0
	b. Crossed by blood vessels	1

### Statistical analysis

One-way analysis of variance (ANOVA) followed by Tukey–Kramer post hoc tests were used to compare the ADC, T2, τ, and Mankin score between ICRS scores. Spearman’s rank correlation was used to identify significant relationships between the ICRS grade and the ADC, T2, τ, and Mankin score. *P* values of < 0.05 were considered to be significant.

## Results

**Fig. 1:**
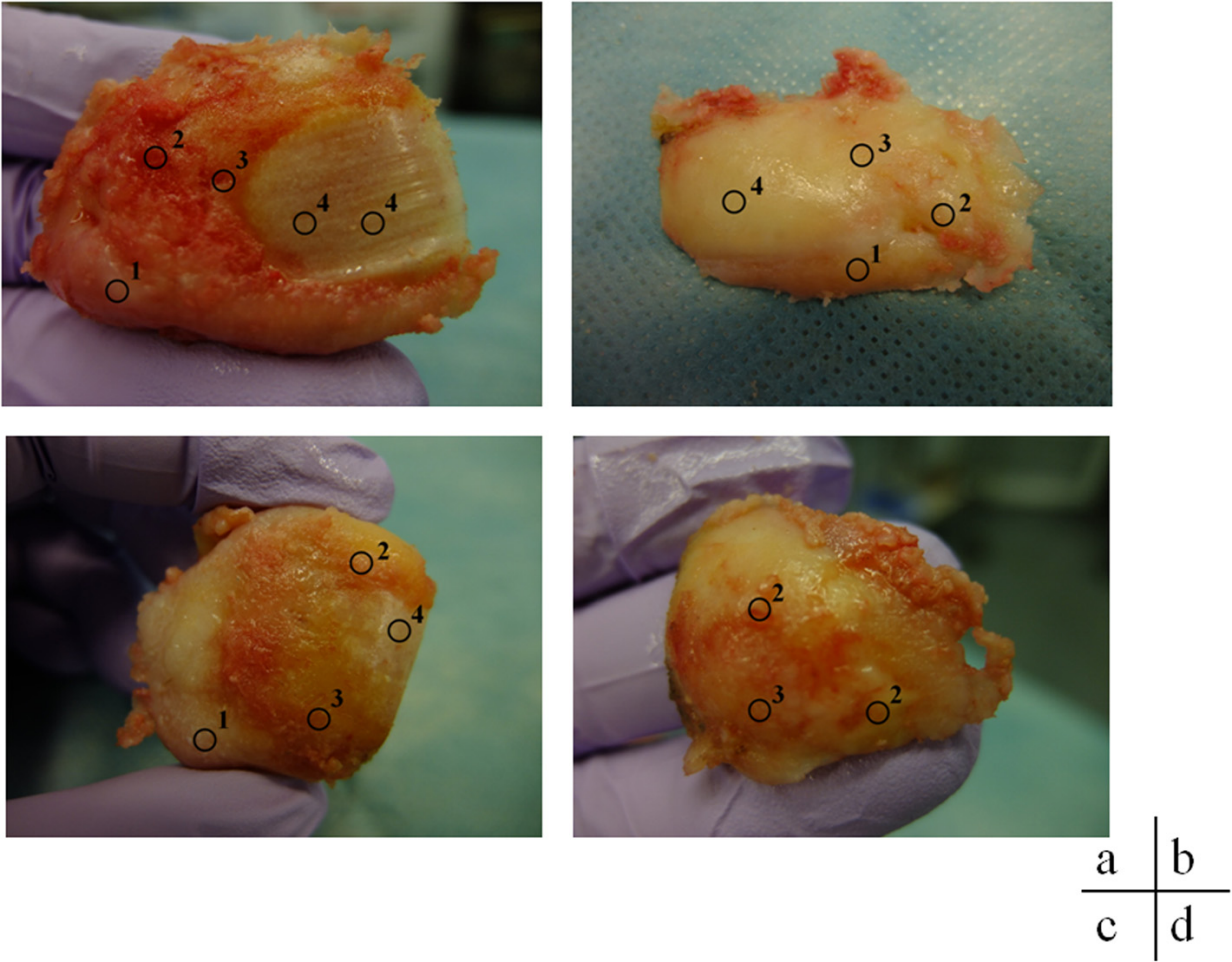
Macroscopic cartilage damage assessment of bone cartilage pieces. The ICRS classification was used to assess the extent of damage to bone cartilage pieces excised during surgery. *ICRS* International Cartilage Repair Society

The grades of each cartilage lesion were grade 1 (*n* = 5), grade 2 (*n* = 8), grade 3 (*n* = 8), and grade 4 (*n* = 8) (Fig. 1, Table 3).

**Table 3:** Details of the cartilage damage

Femoral medial condyle	N	Femoral posterior condyle	n	Tibia	n
Grade 1	0	Grade 1	0	Grade 1	1
Grade 2	2	Grade 2	2	Grade 2	1
Grade 3	2	Grade 3	2	Grade 3	1
Grade 4	2	Grade 4	2	Grade 4	3
Total	6	Total	6	Total	6
Femoral lateral condyle	N	Patella	n		
Grade 1	3	Grade 1	1		
Grade 2	2	Grade 2	1		
Grade 3	1	Grade 3	2		
Grade 4	1	Grade 4	0		
Total	7	Total	4		

**Fig. 2:**
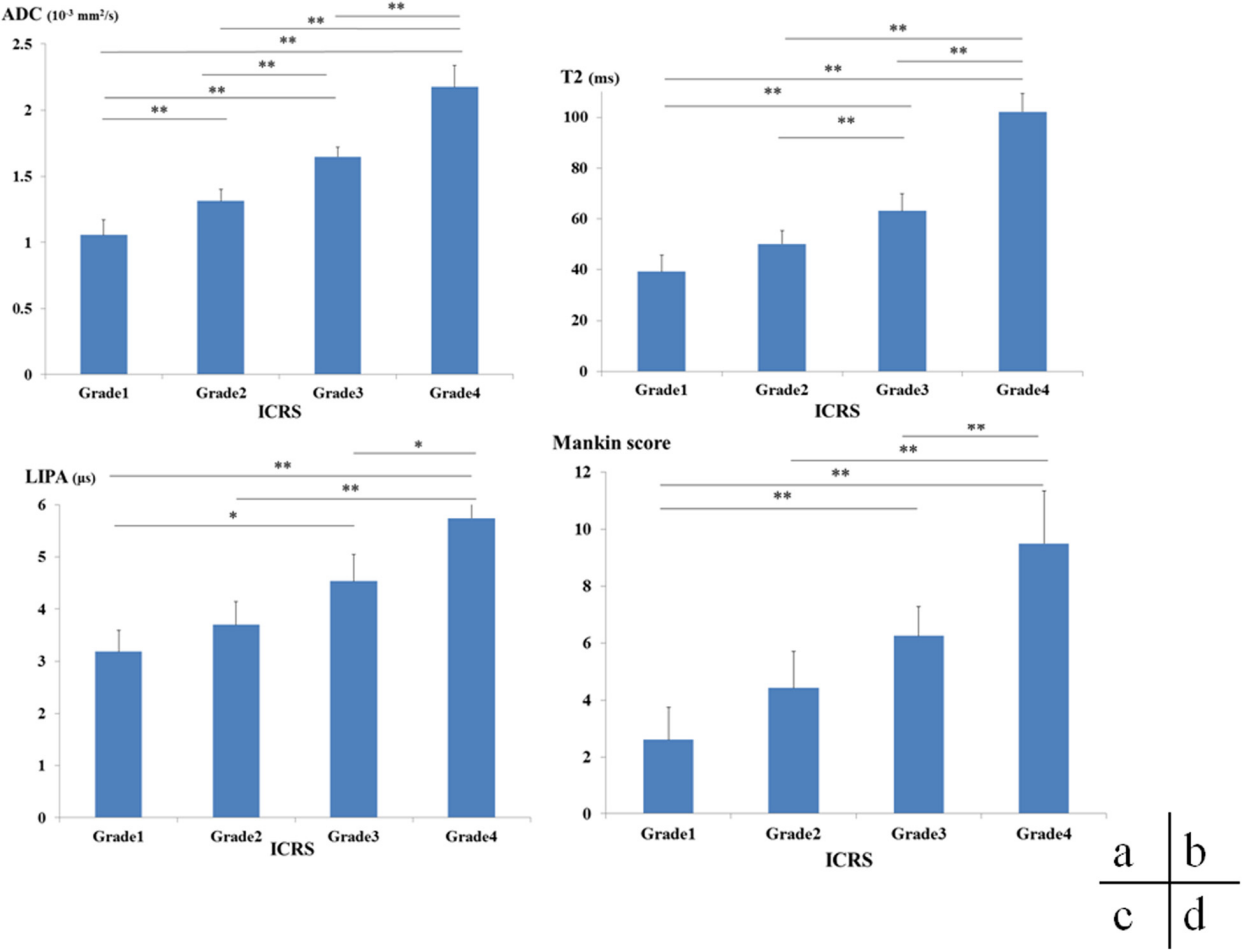
Assessment by ICRS classification and measured values from DT imaging, T2 mapping, LIPA, and Mankin score. **a** Changes in ADC due to cartilage degeneration. The ADC increased as the ICRS grade increased. Statistically significant differences were observed in ICRS grade 1 versus grade 2 (*P* < 0.01); grade 1 versus grade 3 (*P* < 0.01); grade 1 versus grade 4 (*P* < 0.01); grade 2 versus grade 3 (*P* < 0.01); grade 2 versus grade 4 (*P* < 0.01); and grade 3 versus grade 4 (*P* < 0.01). **b** Changes in T2 values due to cartilage degeneration. T2 values increased as the ICRS grade increased. Statistically significant differences were observed in ICRS grade 1 versus grade 3 (*P* < 0.01), grade 1 versus grade 4 (*P* < 0.01); grade 2 versus grade 3 (*P* < 0.01); grade 2 versus grade 4 (*P* < 0.01); and grade 3 versus grade 4 (*P* < 0.01). **c** Changes in τ values due to cartilage degeneration. τ increased as the ICRS grade increased. Statistically significant differences were observed in ICRS grade 1 versus grade 3 (*P* < 0.05); grade 1 versus grade 4 (*P* < 0.01); grade 2 versus grade 4 (*P* < 0.01); and grade 3 versus grade 4 (*P* < 0.05). **d** Changes in Mankin score due to cartilage degeneration. Manikin score increased as ICRS grade increased. Statistically significant differences were observed in ICRS grade 1 versus grade 3 (*P* < 0.01); grade 1 versus grade 4 (*P* < 0.01); grade 2 versus grade 4 (*P* < 0.01); and grade 3 versus grade 4 (*P* < 0.01). *ADC* apparent diffusion coefficient, *DT* diffusion tensor, *ICRS* International Cartilage Repair Society, *LIPA* laser-induced photoacoustic

The ADC: was grade 1 1.06 ± 0.11 × 10^−3^mm^2^/s, grade 2 1.31 ± 0.09 × 10^−3^ mm^2^/s, grade 3 1.65 ± 0.07 × 10^−3^ mm^2^/s ms, and grade 4 2.17 ± 0.17 × 10^−3^ mm^2^/s, with the ADC increasing as the grade increased. Significant differences were observed in grade 1 versus grade 2 (*P* < 0.01), grade 1 versus grade 3 (*P* < 0.01), grade 1 versus grade 4 (*P* < 0.01), grade 2 versus grade 3 (*P* < 0.01), grade 2 versus grade 4 (*P* < 0.01), and grade 3 versus grade 4 (*P* < 0.01) (Fig. 2a).

T2 were: grade 1 39.2 ± 6.50 ms, grade 2 50.1 ± 5.29 ms, grade 3 63.1 ± 6.76 ms, and grade 4 102.2 ± 7.04 ms, with T2 increasing as the grade increased. Significant differences were observed in grade 1 versus grade 3 (*P* < 0.01), grade 1 versus grade 4 (*P* < 0.01), grade 2 versus grade 3 (*P* < 0.01), grade 2 versus grade 4 (*P* < 0.01), and grade 3 versus grade 4 (*P* < 0.01) (Fig. 2b).

τ were: grade 1 3.19 ± 0.40 μs, grade 2 3.70 ± 0.44 μs, grade 3 4.53 ± 0.51 μs, and grade 4 5.75 ± 1.1 μs, with values increasing as the grade increased. Significant differences were observed in grade 1 versus grade 3 (*P* < 0.05), grade 1 versus grade 4 (*P* < 0.01), grade 2 versus grade 4 (*P* < 0.01), and grade 3 versus grade 4 (*P* < 0.05) (Fig. 2c).

**Fig. 3:**
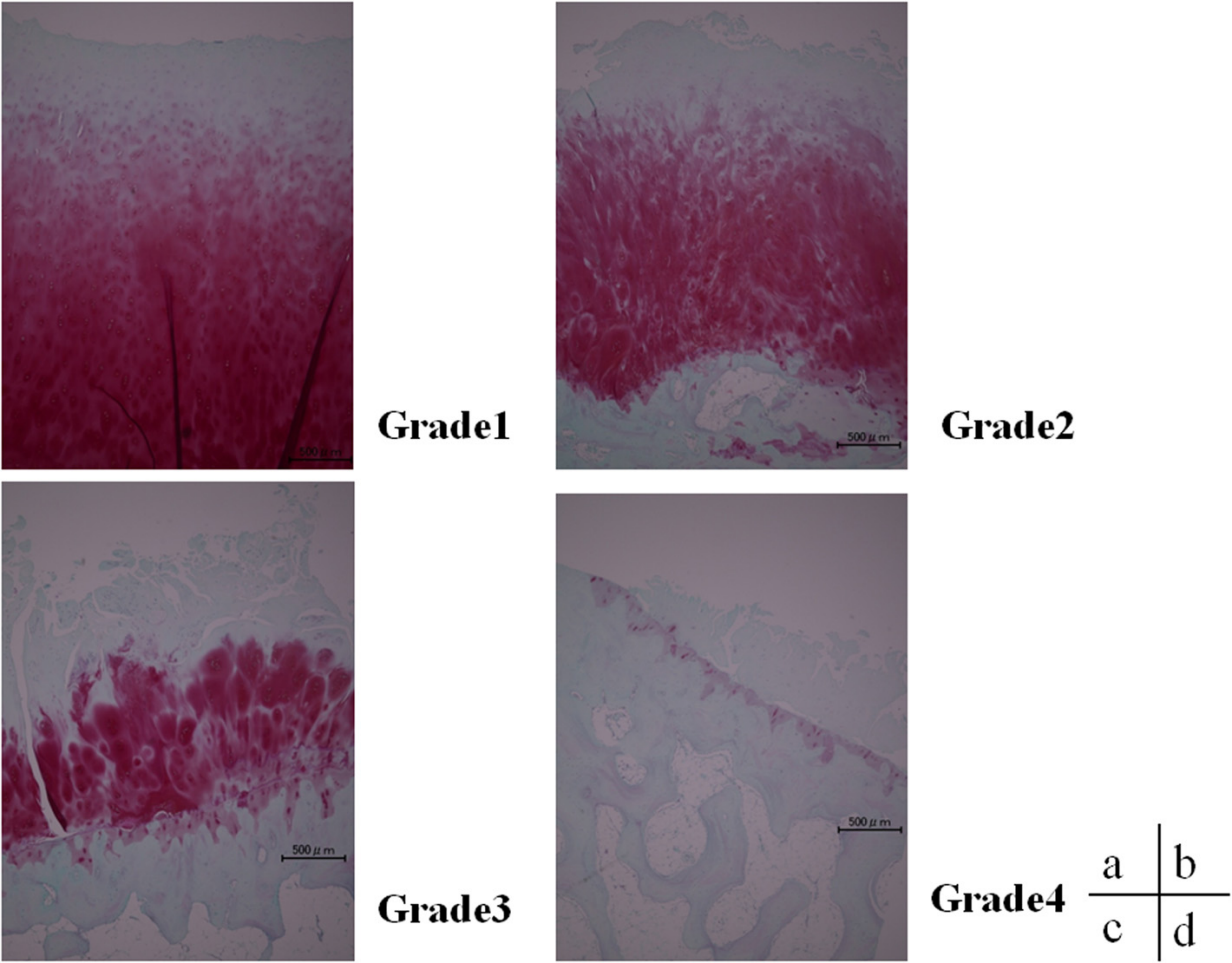
ICRS grade and histological changes. As the ICRS grade advanced, the thinning and decrease in staining of the cartilage layer became increasingly prominent. *ICRS* International Cartilage Repair Society

The Mankin score was: grade 1 2.6 ± 1.14, grade 2 4.43 ± 1.27, grade 3 6.25 ± 1.04, and grade 4 9.5 ± 1.85, with scores increasing as the grade increased. As the grades increased, the degree of histological degeneration progressed (Fig. 3), with significant differences observed in grade 1 versus grade 3 (*P* < 0.01), grade 1 versus grade 4 (*P* < 0.01), grade 2 versus grade 4 (*P* < 0.01), and grade 3 versus grade 4 (*P* < 0.01) (Fig. 2d).

**Fig. 4:**
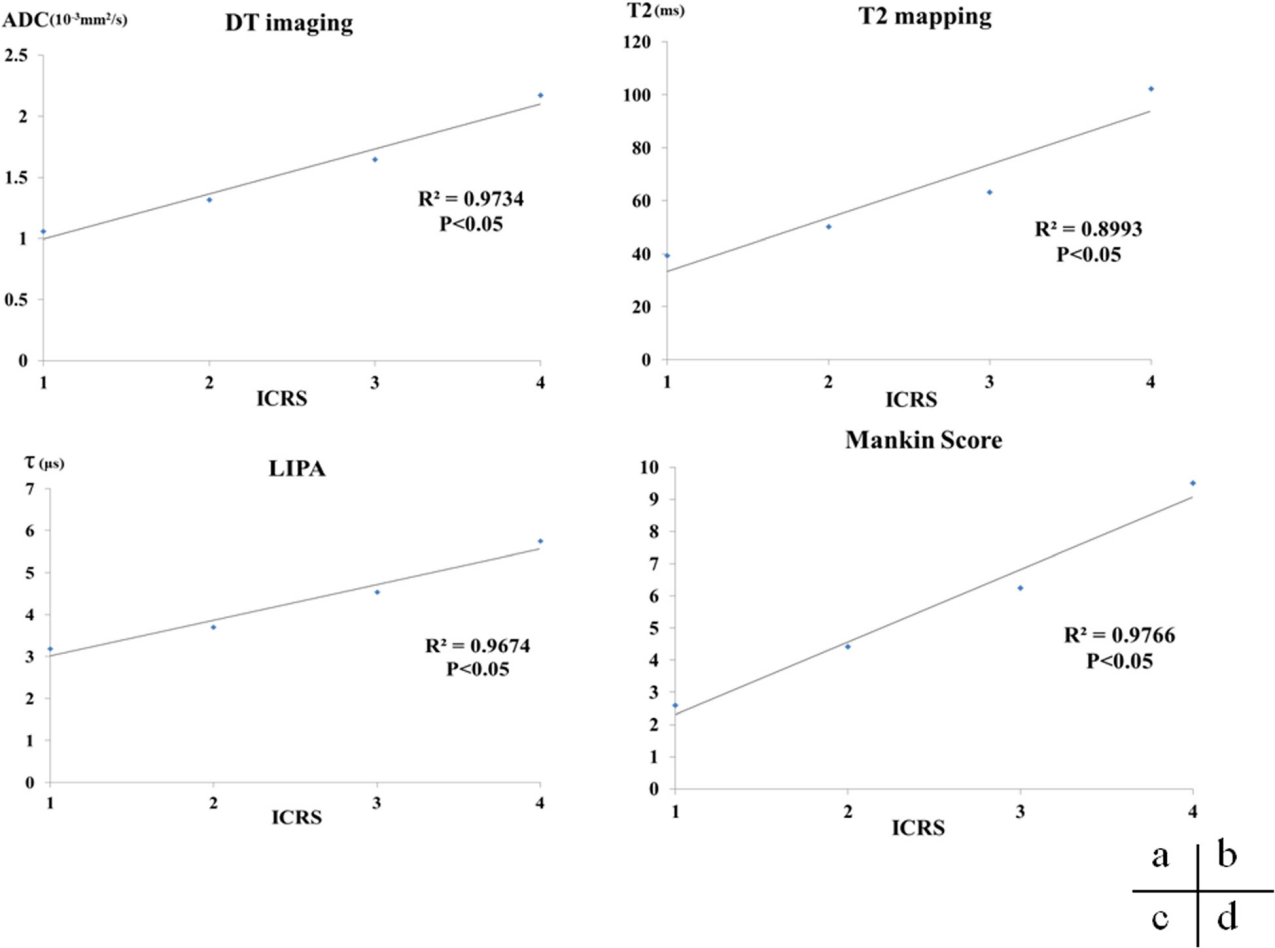
Correlations between ICRS grade and DT imaging, T2 mapping, LIPA, and Mankin score. Strong correlations were observed between ICRS grade and DT imaging (R^2^ = 0.9734), T2 mapping (R^2^ = 0.8993), LIPA (R^2^ = 0.9674), and Mankin score (R^2^ = 0.9766). *DT* diffusion tensor, *ICRS* International Cartilage Repair Society, *LIPA* laser-induced photoacoustic

The DT imaging, T2 mapping, LIPA, and Mankin scores increased as the ICRS grade increased, and correlations were observed in all examinations (Fig. 4).

**Fig. 5:**
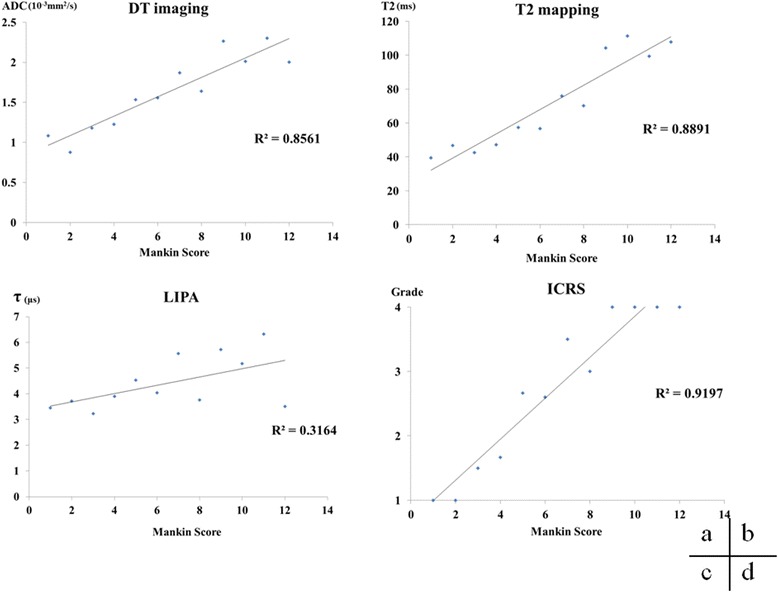
Correlations between Mankin score and DT imaging, T2 mapping, LIPA, and ICRS grade. Although DT imaging, T2 mapping, and ICRS grade showed strong correlations with Mankin score, LIPA had a weaker correlation than MRI with Mankin score. *DT* diffusion tensor, *ICRS* International Cartilage Repair Society, *LIPA* laser-induced photoacoustic

For the degree of histological degeneration, the various methods, and the assessment of ICRS grade, correlations were observed with the Mankin score and DT imaging, T2 mapping, and ICRS grade, but LIPA had a weaker correlation than that for MRI (Fig. 5).

**Fig. 6:**
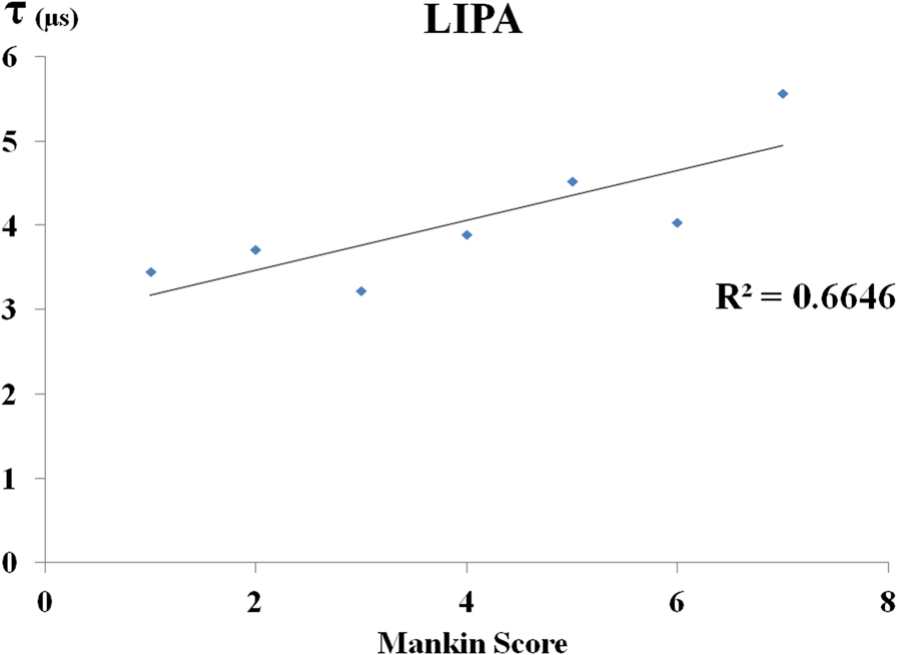
Correlation between moderate histological degeneration and LIPA. Histological degeneration up to moderate in the Mankin score (Mankin score 7 or below) showed a stronger correlation with LIPA than the assessment of the Mankin score as a whole. *LIPA* laser-induced photoacoustic

When correlations were investigated by limiting the Mankin score to no further than moderate histological degeneration, the correlations were stronger than that for the Mankin score overall (Fig. 6).

**Fig. 7:**
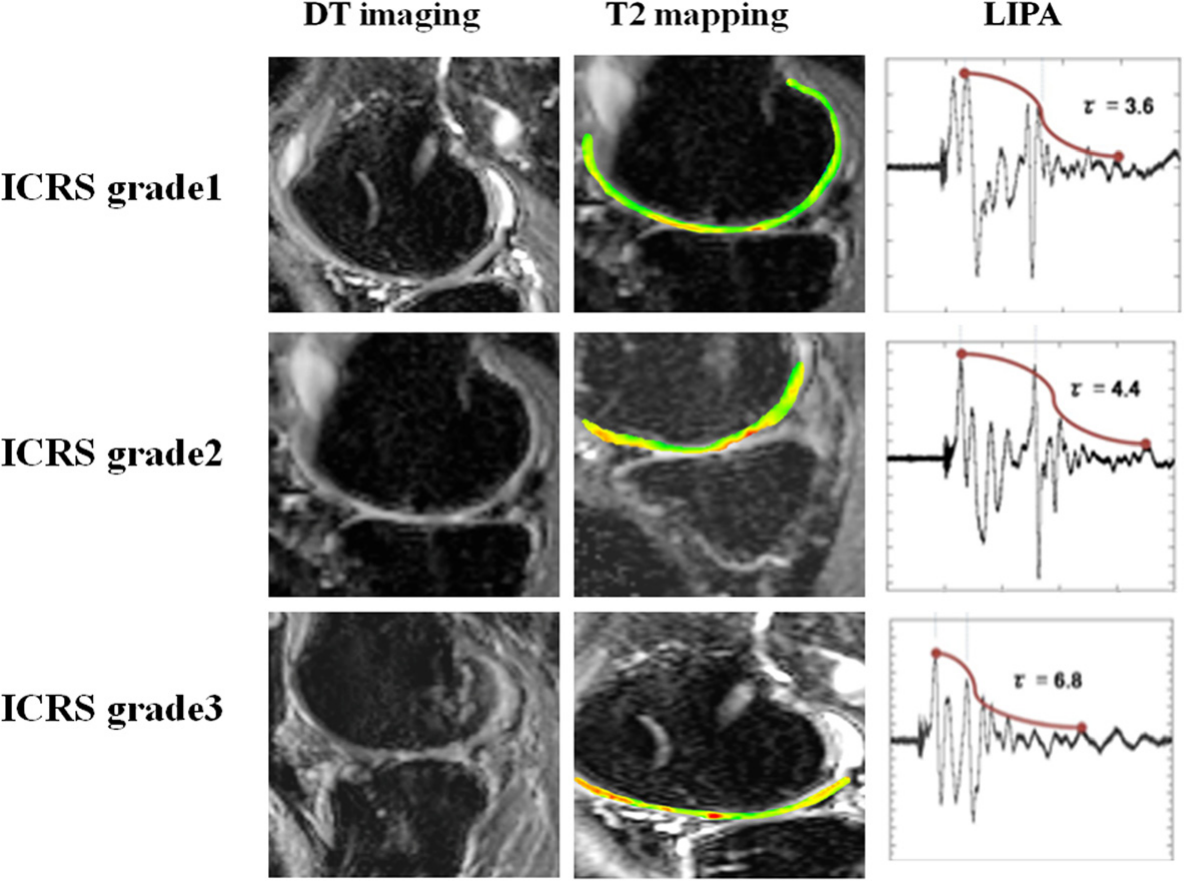
DT imaging, T2 mapping and LIPA of early, moderate and severe cartilage damage. *DT* diffusion tensor, *LIPA* laser-induced photoacoustic (images)

Figure 7 shows the images for some cases of cartilage damage by DT imaging, T2 mapping, and LIPA.

## Discussion

Accurately ascertaining the condition of the cartilage is important for understanding OA of the knee better and for determining the therapeutic effect after surgery. However, for the most part, it has been judged—for example, by the subjective symptoms, osteophyte formation according to X–P, and narrowing of the joint space—that treatment has been aimless and poorly directed. Cartilage assessment by MRI has been widely practiced and includes T2 mapping, which is sensitively reflective of the arrangement of collagen in the cartilage; T1ρ, which is reflective of proteoglycan levels; delayed gadolinium-enhanced MR imaging of cartilage (d-GEMRIC); and 23Na spectroscopic imaging, among others. Recently, cartilage damage has been assessed by DT imaging; among the forms of DT imaging, the ADC has been reflective of the decrease in proteoglycans and in the water content [5], while fractional anisotropy (FA) has captured changes in the arrangement of collagen fibers as anisotropy [5, 26, 29].

Cartilage assessment using the ADC is reportedly useful in both in vitro and in vivo experiments. Meder et al. treated bovine knee joint cartilage with trypsin and performed DT imaging, and they reported that the trypsin treatment group had a more elevated ADC than before treatment [31]. Human articular cartilage also was treated with trypsin and produced results where elevated ADCs were observed [5]. Using the ADC and T2 mapping to assess an OA group and healthy group by X–P, Raya et al. stated that the ADC was effective regarding OA assessment [32]. We confirmed cartilage damage arthroscopically and reported that the ADC made it possible to distinguish between early and advanced cartilage damage [8].

In the results of this study, significant differences were observed between all grades: ICRS grade 1 versus grade 2, grade 1 versus grade 3, grade 1 versus grade 4, grade 2 versus grade 3, grade 2 versus grade 4, and grade 3 versus grade 4. While T2 mapping had a significant difference observed in all specimens for grade 1 versus grade 2, a significant difference was not shown in early cartilage damage. One putative reason that T2 mapping was unable to distinguish early cartilage damage is that in cartilage damage in OA, the decrease in proteoglycans is thought to occur earlier than the decrease in collagen [33], and detection is not possible with T2 mapping as it is sensitively reflective of the arrangement of collagen. An advantage of using MRI to assess cartilage damage is that it can be done noninvasively, but it is often difficult to assess cartilage damage if there is a mixture of OA of different grades. Using MRI, it is impossible to assess either the viscoelastic properties, which represent a fundamental property of the cartilage, or the condition of the tissue.

Ishihara et al. focused on the phenomenon where locally generated stress waves propagate through the tissue and, in so doing, decay because of the innate viscoelasticity of the tissue. Ishihara et al. developed a technique where the condition of the tissue and the mechanical properties of the articular cartilage itself are assessed by fluorescence information and photoacoustic signals are obtained noninvasively using nanosecond-pulsed laser [11–22]. The nanosecond-pulsed laser used with LIPA has no impact on the potential for cell proliferation and can be used both safely and precisely, even though the cartilage is irradiated with 50 times the amount typically used [10, 15]. In assessing the viscoelastic properties by LIPA under arthroscope, the ability to assess the properties of the cartilage without having to collect tissue is extremely useful. Thus far, in a cartilage degeneration group treated by trypsin, using LIPA, it was reported that as τ extension or culture time increases, τ decreases [10, 12, 15].

In our LIPA results, significant differences were observed in ICRS grade 1 versus grade 3, grade 1 versus grade 4, grade 2 versus grade 4, and grade 3 versus grade 4, and results identical to the Mankin score for histological assessment were obtained (Fig. 2). Although significant differences were not observed in early cartilage damage, significant differences were observed when there was moderate or higher damage, making it useful for cartilage damage assessment. The fact that LIPA produced results identical to the histological findings is believed to be because cartilage damage reduced the extracellular matrix and lowered the viscoelasticity of the cartilage, thus producing an observed extension of the relaxation time in LIPA. LIPA has been proven to offer examination equivalent to histological assessment without the need to collect tissue.

Our results here support the in vitro results obtained previously, and it is believed to be a practical method that could be used during actual surgery. However, no correlation was observed in a comparison with the Mankin score overall. Therefore, we separated the Mankin scores up to 7, which is up to moderate damage, and noted a correlation with LIPA (Fig. 6), at which point a correlation was observed between the Mankin score and LIPA. In advanced cartilage damage, the cartilage layer has often completely disappeared, and it may be difficult to make assessments with LIPA that measures the viscoelastic properties of the cartilage. Therefore, LIPA makes macroscopic assessment available with cartilage damage of any ICRS grade, but histologically, it has been a useful examination for up to moderate histological degeneration (Mankin score 7 or below).

We found that cartilage damage could be evaluated using DT imaging, T2 mapping, or LIPA. An advantage of MRI is that it enables noninvasive assessment of cartilage damage, but evaluation using this method is difficult if there are several localized areas of cartilage damage of varying grades. Although LIPA requires an invasive procedure, including arthroscopy, it enables evaluation of cartilage property at different damaged sites without the need for tissue sampling; therefore, LIPA is valuable for purposes such as assessment of cartilage damage during surgery and also cartilage regeneration as a follow-up. Thus, both MRI and LIPA have advantages and disadvantages for the evaluation of cartilage, and rather than using only one of these techniques, it is desirable to use both in combination, depending on the state of OA and the patient's condition.

## Conclusions

In our study, ADC enables assessment in all stages of cartilage damage and T2 mapping enables assessment of moderate or higher cartilage damage. τ measurement by LIPA produces viscoelasticity ratios and enables assessment of up to moderate cartilage damage. In the assessment of knee OA, it is sometimes difficult to assess damaged cartilage with MRI alone, and we feel that it is desirable to make use of LIPA and MRI combined.

## Abbreviations

τ: relaxation time
ADC: Apparent diffusion coefficient
d-GEMRIC: Delayed gadolinium-enhanced MRI of cartilage
DT: Diffusion tensor
EPI: Echo planar imaging
FA: Fractional anisotropy
FOV: Field of view
ICRS: International Cartilage Repair Society
LIPA: Laser-induced photoacoustic
MRI: Magnetic resonance imaging
NEX: Number of excitations
OA: Osteoarthritis
ROI: Region of interest
TE: Echo time
TKA: Total knee arthroplasty
TR: Repetition time
WFS: Water-fat shift
